# Weight retention at six weeks postpartum and the risk of gestational diabetes mellitus in a second pregnancy

**DOI:** 10.1186/s12884-019-2423-3

**Published:** 2019-08-01

**Authors:** Jing Liu, Guang Song, Tao Meng, Ge Zhao, Songning Guo

**Affiliations:** 1 Department of Obstetrics, The First Affiliated Hospital of China Medical University, No. 155 Nanjing North Street, Heping District, Shenyang, 110001 Liaoning Province China; 2 Department of Ultrasound, Shengjing Hospital of China Medical University, Shenyang, China

**Keywords:** Gestational diabetes mellitus, Postpartum weight retention, Interpregnancy BMI changes

## Abstract

**Background:**

Gestational diabetes mellitus (GDM) is a common cause of maternal morbidity, and can lead to the development of diabetes later in life. Pre-pregnancy body weight is associated with the change in body mass index (BMI) between a first and second pregnancy. Compared with long-term change in BMI between pregnancies, the most accessible follow-up point to investigate BMI change is 6 weeks after the initial pregnancy. The present study aimed to assess the association between weight retention at 6 weeks postpartum and the risk of GDM in a subsequent pregnancy.

**Methods:**

We recruited 6429 singleton pregnancies into this retrospective cohort study. For each pregnancy, we calculated weight retention at 6 weeks postpartum after the first pregnancy, the interpregnancy BMI change between pregnancies, and the gestational weight gain in the second pregnancy. Risk was represented by the odds ratio (OR) and 95% confidence intervals (CIs). We then determined the relationship between postpartum weight retention at 6 weeks after the initial pregnancy, and the interpregnancy change in BMI between pregnancies. Analyses were stratified by BMI during the first pregnancy.

**Results:**

Compared to women with a stable BMI (− 1 to 1), interpregnancy BMI gains were associated with an increased risk of GDM in the second pregnancy. Risk increased significantly for women with a BMI below and above 25 during the first pregnancy, although the increase was greater in the women with a BMI < 25. The risk of GDM in the second pregnancy was higher in women with inadequate weight gain during the second gestation. The weight retention at 6 weeks postpartum, where there was a gain of > 3 BMI units was significantly more related to weight gain more than when there was 1 BMI unit gain between pregnancies (*P* < 0.05) and associated with an increased incidence of GDM in the second pregnancy (OR = 2.95, 95% CI: 1.95 ~ 4.45). Women who showed a change in BMI that was > 3 units at 6 weeks postpartum after the first pregnancy showed an increased risk for BMI subsequently (OR = 1.42, 95% CI: 1.08~1.87).

**Conclusions:**

Women who gained more than 3 BMI units at 6 weeks postpartum were associated with an increased risk of BMI in a subsequent pregnancy. Six weeks postpartum provides a new early window of opportunity to identify risk factors for a subsequent pregnancy and allows us to implement primary prevention strategies.

## Background

Gestational diabetes mellitus (GDM) is a condition in which carbohydrate intolerance develops during pregnancy; this is a common cause of maternal morbidity and can lead to the development of diabetes in later life [1]. The offspring of women with GDM are at an increased risk of macrosomia, neonatal hypoglycemia, hyperbilirubinemia, shoulder dystocia, and birth trauma. Being overweight and obesity before and during pregnancy increases the risk of GDM [2–5].

The increasing global prevalence of obesity has prompted the World Health Organization (WHO) to designate obesity as one of the most important threats to global health [6]. Understanding the association between GDM and the current obesity epidemic among pregnant women could help us establish effective and appropriate preventative strategies with which to improve maternal-fetal outcomes. Previous studies have demonstrated that pre-pregnancy weight gain increases the risk of GDM [7–9]. Weight gain during pregnancy has also been showed to associate with GDM [10]. Pre-pregnancy body weight is associated with the change in body mass index (BMI) between a first and second pregnancy. Some researchers have reported that weight gain between two consecutive pregnancies increased the risk for GDM in the second pregnancy [11, 12]. The increase in BMI from the first to the second pregnancy could represent postpartum weight retention (PPWR). PPWR is the weight gain related to the pregnancy [13], including short-term retention (4–6 weeks) [14] and long-term retention [15]. Mean estimates of PPWR are small, ranging from − 0.27 kg to 3 kg [16]. However, a substantial subgroup of women retain considerable amounts of weight postpartum [17–19]. Many international clinical guidelines recommend that overweight and obese women should lose weight prior to a second pregnancy in order to reduce the risk of adverse pregnancy outcomes [20, 21]. Therefore, it is highly beneficial to identify women who are at risk for significant PPWR because this could help to reduce the risk of adverse outcomes in the subsequent pregnancy. Previous studies have already shown the PPWR between pregnancies is a risk factor for GDM in a second pregnancy [11, 12]. However, it is meaningless to inform women that they have a risk for GDM when they have already attained a second pregnancy while overweight or obese. We therefore need to identify high-risk women at early timepoint and implement primary preventative strategies.

There are only three times during the life of a woman when she regularly accesses the health care system and is seen by a trained health care provider: as an infant, for postpartum care and pregnancy, and when she develops a chronic disease. The period of postpartum care could help doctors to identify the high-risk women who need be closely monitored in later life. The aim of this study was to estimate the association between PPWR 6 weeks after the initial pregnancy and the risk of developing GDM during a second pregnancy. We also evaluated the relationship between an interpregnancy change in BMI between pregnancies, weight changes during the second pregnancy and the risk for GDM in a second pregnancy.

## Methods

### Study design, setting and population

The study, which was carried out between 2011 and 2018, featured a retrospective cohort study design and was conducted among Asian women who gave birth to their second singletons between 28 and 42 weeks of gestation at the Department of Obstetrics. GDM was defined according to the International Association of Diabetes and Pregnancy Study Groups; a diagnosis of GDM was made when one or more of the test parameters equaled or exceeded the following cut points: fasting 5.1 mmol/L, 1-h 10.0 mmol/L, or 2-h 8.5 mmol/L [22]. All participants provided written informed consent and the study protocol was approved by the Medical Ethics Review Board of China Medical University (Shenyang, Liaoning, China). To evaluate the first-time occurrence of GDM in the second pregnancy, we excluded women who experienced GDM in the first pregnancy.

### Data collection and measurements

First, we calculated the BMI of all pregnant woman (weight/height^2^, kg/m^2^). For each pregnancy, pre-pregnancy weight was recorded in the maternal prenatal records at the first visit. We defined the difference between the pre-pregnancy BMI of the first pregnancy and the second pregnancy as ‘the interpregnancy BMI change’. The weight at 6 weeks postpartum was also recorded in their records. We excluded any women who could not provide their prenatal records for their first and second births. The weight retention at 6 weeks postpartum after the first pregnancy was defined as the BMI at 6 weeks after the first birth minus the prepregnant BMI of the first pregnancy. Maternal weight was recorded either during the last prenatal visit or before delivery. We used IOM (Institute of Medicine) guidelines to assess gestational weight gain in the second pregnancy because this is the most clinically relevant categorization of weight gain and is simply classified as inadequate, adequate, or excessive. Adequate weight gain was defined using gestational age-based guidelines rather than the total expected weight gain to account for the shorter time interval for weight gain in the event of preterm delivery [23]. Adequate gestational weight gain was based on BMI class in the third trimesters as follows: underweight 1–1.3 pound/week, normal weight 0.8–1 pound/week, overweight 0.5–0.7 pound/week, and obese 0.4–0.6 pound/wk. The total gestational weight gain at the gestational age of delivery was categorized based on the recommended gestational weight gain range for that gestational week: recommended weight gain if gestational weight gain was within the recommended range, excessive weight gain if gestational weight gain was greater than the upper bound of this range, inadequate weight gain if gestational weight gain was between 0 and lower bound of this range, or weight loss. Interpregnancy interval was calculated as the number of complete weeks between the birth of the first and the second child minus the duration of the second pregnancy.

We further explored the association between BMI change and the risk of GDM in a second pregnancy by using the prepregnant BMI in the first pregnancy (BMI < 25 kg/m^2^ versus ≥25 kg/m^2^) in stratified analyses. We categorized BMI change as less than − 1 (a loss in BMI more than 1 unit), − 1 to less than 1 (the reference group), 1 to less than 2, 2 to less than 3, and 3 or more BMI units; this system has been used in several other relevant publications [24, 25].

### Statistical analysis

Continuous parameters were expressed as the mean ± standard deviation. Differences of continuous parameters (maternal age second pregnancy, interpregnancy interval and BMI (kg/m^2^) in the first pregnancy) between the GDM and non-GDM groups (and shown in Table 1) were analyzed using the independent-samples *t* test. Differences of categorial parameters (interpregnancy BMI change, gestational weight gain during the second pregnancy and weight retention at 6 weeks postpartum after the first pregnancy) between the GDM and non-GDM groups (and shown in Table 1**)** were analyzed using Pearson’s chi-squared test. General linear models were used to estimate the association between GDM in the second pregnancy, interpregnancy interval, BMI in the first pregnancy, interpregnancy BMI change, gestational weight gain during the second pregnancy, and change in BMI at 6 weeks postpartum after the first pregnancy.

**Table 1:** Clinical characteristics in the second pregnancy associated with GDM status

	GDM in the second pregnancy		*P*
	Yes (*n* = 1098)	No (*n* = 5331)	
Maternal age (y) second pregnancy	32.7 ± 4.7	32.7 ± 4.1	0.533
Interpregnancy interval (week)	47.5 ± 26.1	43.3 ± 27.1	< 0.001
BMI (kg/m^2^) in the first pregnancy	22.9 ± 3.3	22.5 ± 4.2	0.008
Interpregnancy BMI change			< 0.001
Lost 1.0 or more	31	503	
Remain stable (±1.0)	476	2852	
Gained 1.1–2.0	237	812	
Gained 2.1–3.0	159	542	
Gained more than 3.0	195	622	
Gestational weight gain during the second pregnancy			< 0.001
Inadequate	485	1745	
Adequate	365	2165	
Excessive	248	1421	
The weight retention at six weeks postpartum after the first pregnancy			< 0.001
Lost 1.0 or more	3	15	
Remain stable (±1.0)	27	218	
Gained 1.0–1.9	181	1060	
Gained 2.0–2.9	260	2227	
Gained 3.0 or more	627	1811	

*BMI*, Body mass index; *GDM*, Gestational diabetes mellitus; *OR*, Odds ratio

When calculated the odds ratio (OR) and 95% confidence intervals (CIs) and used a stable interpregnancy BMI change (±1.0), adequate gestational weight gain during the second pregnancy and stable BMI change at 6 weeks postpartum after the first pregnancy as a reference group in Table 2–5. When calculating the adjusted OR, we used parameters which showed statistical changes when compared between the GDM group and the control group according to the results of Table 1. In order to evaluate the effect of BMI in the first pregnancy, we used the subgroup/stratified analysis and Logistic analysis (BMI *<* 25 and BMI ≥ 25) (Tables 2, 3, 4).

**Table 2:** Odds ratios of interpregnancy BMI change associated with GDM in the second pregnancy

	Number of GDM in the second pregnancy	Unadjusted OR (95% CI)	Adjusted OR (95% CI)^*#^
All women^*^			
Interpregnancy BMI (kg/m^2^) change			
Lost 1.0 or more	31	0.36(0.25~0.54)	0.28 (0.19~0.43)
Remain stable (±1.0)	476	1.00	1.00
Gained 1.0–1.9	237	1.75 (1.47~2.08)	1.77 (1.49~2.11)
Gained 2.0–2.9	159	1.76 (1.44~2.15)	1.79 (1.46~2.19)
Gained 3.0 or more	195	1.88 (1.56~2.27)	1.80 (1.49~2.17)
BMI in the first pregnancy < 25^#^			
Interpregnancy BMI change			
Lost 1.0 or more	26	1.05 (0.68~1.61)	1.01 (0.66~1.56)
Remain stable (±1.0)	375	1.00	1.00
Gained 1.0–1.9	183	1.82 (1.50~2.22)	1.89 (1.55~2.31)
Gained 2.0–2.9	113	1.84 (1.46~2.34)	1.91 (1.50~2.42)
Gained 3.0 or more	140	1.97 (1.58~2.45)	1.93 (1.55~2.41)
BMI in the first pregnancy ≥25^#^			
Interpregnancy BMI change			
Lost 1.0 or more	5	0.08 (0.03~0.19)	0.08 (0.03~0.19)
Remain stable (±1.0)	101	1	1
Gained 1.0–1.9	54	1.50 (1.04~2.17)	1.51 (1.04~2.18)
Gained 2.0–2.9	46	1.51 (1.02~2.23)	1.51 (1.02~2.23)
Gained 3.0 or more	55	1.61 (1.11~2.33)	1.62 (1.12~2.34)

*BMI*, Body mass index; *GDM*, Gestational diabetes mellitus; *OR*, Odds ratio

^*^ Model adjusted for interpregnancy interval, BMI in the first pregnancy, gestational weight gain during 2nd pregnancy, and BMI change at six weeks postpartum after the first pregnancy

^#^ Model adjusted for interpregnancy interval, gestational weight gain during 2nd pregnancy, and BMI change at six weeks postpartum after the first pregnancy

**Table 3:** Odds ratios of gestational weight gain during second pregnancy associated with GDM

	Number of GDM in the second pregnancy	Unadjusted OR (95% CI)	Adjusted^*#^				
			OR (95% CI)	β value	SE	Wald χ2	*P*
All women^*^							
Gestational weight gain during the second pregnancy							
Inadequate	485	1.65(1.41~1.91)	1.67(1.43~1.93)	0.51	0.08	44.49	< 0.001
Adequate	365	1	1	/	/	/	/
Excessive	248	1.04(0.87~1.23)	1.04(0.88~1.24)	0.05	0.09	0.26	0.610
BMI (kg/m^2^) in the first pregnancy < 25^#^							
Gestational weight gain during the second pregnancy							
Inadequate	374	1.69(1.42~2.00)	1.69(1.42~2.01)	0.53	0.09	35.61	< 0.001
Adequate	274	1	1	/	/	/	/
Excessive	189	1.06(0.87~1.30)	1.09(0.89~1.34)	0.09	0.10	0.71	0.401
BMI in the first pregnancy ≥25^#^							
Gestational weight gain during the second pregnancy							
Inadequate	111	1.53(1.13~2.07)	1.54(1.14~2.09)	0.43	0.16	7.72	0.005
Adequate	91	1	1	/	/	/	/
Excessive	59	0.96(0.67~1.36)	0.94(0.66~1.34)	−0.06	0.18	0.10	0.752

BMI, body mass index; GDM, gestational diabetes mellitus; OR, odds ratio; SE, standard error

^*^ Model adjusted for interpregnancy interval, BMI in the first pregnancy, interpregnancy BMI change, and BMI change at six weeks postpartum after the first pregnancy

^#^ Model adjusted for interpregnancy interval, interpregnancy BMI change, and BMI change at six weeks postpartum after the first pregnancy

**Table 4:** Odds ratios of BMI change at six weeks postpartum after the first pregnancy associated with GDM in the second pregnancy

	Number of GDM in the second pregnancy	Unadjusted OR (95% CI)	Adjusted OR (95% CI)^*#^
All women^*^			
The weight retention at six weeks postpartum after the first pregnancy			
Lost 1.0 or more	3	1.61(0.44~5.94)	1.53(0.41~5.63)
Remain stable (±1.0)	27	1	1
Gained 1.0–1.9	181	1.38(0.90~2.12)	1.43(0.93~2.20)
Gained 2.0–2.9	260	0.94(0.62~1.43)	1.00(0.66~1.53)
Gained 3.0 or more	627	2.80(1.86~4.21)	2.95(1.95~4.45)
BMI in the first pregnancy < 25^#^			
BMI change at six weeks postpartum after the first pregnancy			
Lost 1.0 or more	2	2.21(0.43~11.40)	2.29(0.44~11.78)
Remain stable (±1.0)	20	1	1
Gained 1.0–1.9	135	1.40(0.85~2.32)	1.41(0.85~2.32)
Gained 2.0–2.9	205	0.98(0.60~1.60)	0.99(0.61~1.62)
Gained 3.0 or more	475	2.71(1.68~4.37)	2.73(1.69~4.41)
BMI in the first pregnancy ≥25^#^			
BMI change at six weeks postpartum after the first pregnancy			
Lost 1.0 or more	1	1.13(0.12~10.37)	1.12(0.12~10.36)
Remain stable (±1.0)	7	1	1
Gained 1.0–1.9	46	1.31(0.57~3.04)	1.31(0.57~3.04)
Gained 2.0–2.9	55	0.81(0.35~1.85)	0.81(0.35~1.86)
Gained 3.0 or more	152	3.03(1.36~6.75)	3.02(1.35~6.76)

*BMI*, Body mass index; *GDM*, Gestational diabetes mellitus; *OR*, Odds ratio

^*^ Model adjusted for interpregnancy interval, BMI in the first pregnancy, interpregnancy BMI change, and gestational weight gain during 2nd pregnancy

^#^ Model adjusted for interpregnancy interval, interpregnancy BMI change, and gestational weight gain during 2nd pregnancy

**Table 5:** Odds ratios of the weight retention at six weeks postpartum after the first pregnancy associated with interpregnancy BMI change

	Interpregnancy BMI change		Unadjusted OR (95% CI)	Adjusted OR (95% CI)
	< 1	≥1		
The weight retention at six weeks postpartum after the first pregnancy				
Lost 1.0 or more	8	10	2.23(0.85~5.88)	2.23(0.89~6.13)
Remain stable (±1.0)	157	88	1	1
Gained 1.0–1.9	773	468	1.08(0.81~1.44)	1.07(0.80~1.42)
Gained 2.0–2.9	1579	908	1.03(0.78~1.35)	1.00(0.76~1.32)
Gained 3.0 or more	1345	1093	1.45(1.10~1.90)	1.42(1.08~1.87)

*BMI*, Body mass index; *OR*, Odds ratio

Model adjusted for interpregnancy interval, BMI in the first pregnancy, gestational diabetes mellitus, and gestational weight gain during the second pregnancy

A two-tailed *P* < 0.05 was used to define statistical significance. Statistical analysis was performed using STATA version 14.0 software.

## Results

A total of 6429 mothers met our eligibility criteria and were included in our final analysis. The overall prevalence of GDM during a second pregnancy was 17.1% (1098/6429). Table 1 shows that the difference between the two groups in terms of maternal age at the second pregnancy was not statistically significant. The interpregnancy interval increased from 43.0 weeks in the control group to 47.5 weeks in the GDM group. The BMI in the first pregnancy increased from 22.5 in the control group to 22.9 in the GDM group. The difference between the two groups in terms of interpregnancy change in BMI was statistically significant. Inadequate gestational weight gain during the second pregnancy was associated with a higher incidence [21.7% (485/485 + 1745)] of GDM; this compared to 14.4% (365/365 + 2165) in the adequate group and 14.9% (248/248 + 1421) in the excessive group. The difference between the two groups in terms of gestational weight gain during the second pregnancy was statistically significant, as was weight retention at 6 weeks postpartum after the first pregnancy.

Table 2 shows the OR for GDM during the second pregnancy according to inter-pregnancy BMI. Compared to the subjects who maintained a stable BMI, the risk during the second pregnancy was higher in subjects who showed a gain in BMI units. The risk increased obviously when BMI units gaining more. OR increased from 1.00 to 1.77 (95% CI: 1.49~2.11), 1.79 (95% CI: 1.46~2.19), 1.80 (95% CI: 1.49~2.17) as BMI units were gained between pregnancies. Stratified analysis showed that the risk was also aggravated when inter-pregnancy weight increased and was especially obvious in subjects who had a pre-pregnant BMI < 25 in the first pregnancy (OR = 1.93, 95% CI: 1.55~2.41). Stratified analysis also showed that the risk reduced in subjects who had a BMI > 25 during the first pregnancy and lost more than 1 BMI unit (OR = 0.08, 95% CI: 0.03~0.19).

Table 3 shows the adjusted OR for GDM in the second pregnancy according to gestational weight gain during the second pregnancy. The OR of GDM in women with an inadequate weight gain during the second pregnancy was 1.69 (95% CI: 1.42 ~ 2.01) in women with a BMI < 25 in the first pregnancy, and was 1.54 (95% CI: 1.14 ~ 2.09) in women with a BMI > 25 in the first pregnancy. The risk of GDM in the second pregnancy was higher in women with an inadequate weight gain during the second pregnancy, regardless of whether they were overweight or obese in their first pregnancy. The effect of inadequate weight gain on GDM was not related to BMI in the first pregnancy. There was no evidence to suggest that excessive gestational weight gain during the second pregnancy could either increase or decrease the risk of GDM.

Table 4 shows the adjusted OR for GDM in the second pregnancy according to weight retention at 6 weeks postpartum of the first pregnancy. A weight retention at 6 weeks which was associated with a gain of > 3 BMI units led to an increase in the incidence of GDM in the second pregnancy (OR = 2.95, 95% CI: 1.95 ~ 4.45 for all women; OR = 2.73, 95% CI: 1.69 ~ 4.41 for women who had a BMI < 25 in the first pregnancy; OR = 3.02, 95% CI: 1.35 ~ 6.76 for women who had a BMI > 25 in the first pregnancy).

The association between short-term weight retention at 6 weeks postpartum and long-term retention, which refers to the interpregnancy weight change between the first and second pregnancy is shown in Table 5. Women who showed a change in BMI of more than 3 units at 6 weeks postpartum of the first pregnancy were associated with an increased risk for BMI gain in later life (OR = 1.42, 95% CI: 1.08 ~ 1.87).

## Discussion

Our findings suggest that PPWR at 6 weeks after the initial pregnancy, interpregnancy BMI change between pregnancies, and gestational weight gain in the second pregnancy are all independently associated with GDM in the second pregnancy.

Our current research confirmed that women who gained BMI units between two consecutive pregnancies have an increased risk for GDM in their second pregnancy; this is consist with the findings of previous studies [9, 11, 12]. Although a gain in BMI increased the risk of GDM for all women, significantly stronger association were evident for women with a normal BMI in their first pregnancy as compared to overweight or obese women. While we didn’t classify the BMI change < − 2 as a group, as in other studies [5, 10], we still identified that a loss of BMI units between pregnancies represented a protective factor for the risk of GDM in the second pregnancy in women who were overweight or obese. These results suggest that overweight/obese women should lose weight postpartum in order to avoid GDM in a subsequent pregnancy. In contract to the relationship between interpregnancy BMI change and GDM, we also found that insufficient weight gain during the second pregnancy was related to a higher risk of GDM in a subsequent pregnancy, regardless of the first pre-pregnancy BMI. This may be due to the fact that women with GDM are closely supervised and undergo strategies for life modification, which may result in lower gestational weight gain during the second pregnancy.

GDM is characterized by decreased insulin sensitivity and an insufficient insulin response [26]. There is an adaptive physiologic state of decreased insulin sensitivity necessary for supplying sufficient energy to support fetal development during pregnancy. Insulin sensitivity decreases by 50–60% under the normal physiological state during pregnancy [27, 28]. Most women are able to maintain normal glucose tolerance by increasing their insulin secretion to compensate for this insulin resistant state [26]. However, women with a normal weight generally have higher insulin sensitivity than obese and overweight women [28] A subclinical reduction in insulin sensitivity can be caused by weight gain between a first and second pregnancy. Because women with a normal weight are used to higher insulin sensitivity, they are more susceptible to develop GDM due to their reduction in insulin sensitivity during pregnancy [28].

Pregnancy is one of the few occasions in which young women access the healthcare system on a regular basis, the providing time for appropriate assessment [29, 30]. Six weeks postpartum represents the end of pregnancy. By 6 weeks after delivery, most of the changes associated with pregnancy, labor and delivery will have resolved and the body will has reverted to the nonpregnant state [31, 32]. We should therefore assess changes in BMI at 6 weeks postpartum because women need to evaluate their recovery after delivery; after this timepoint, those women will be lost to follow-up until they become pregnant again. However, it is too late to prevent the effect of interpregnancy weight change on GDM when women are already pregnant. Our study showed that the women who gained more than 3 BMI units at 6 weeks postpartum were associated with a gain BMI in their later life and an increased risk of GDM in subsequent pregnancy. Compared to the short-term (4–6 weeks) retention, a change in BMI between pregnancies is affected by the frequency of physical exercise, food intake, and the duration of breastfeeding [33]. Most women showing an increased BMI at 6 weeks after an initial pregnancy will proceed to achieve a stable BMI in their future life via apropriate modification to their lifestyle. However, excessive PPWR is an important contributor to obesity in the maternal future life [16, 25]. Moreover, women who gain more weight during pregnancy than the US IOM recommendations also retain more weigh postpartum than mothers whose weight gain is compliant with IOM recommendations [33, 34]. Although our study did not investigate the relationship between weight gain in the first pregnancy and GDM in the second pregnancy, as with the study reported by Bogaerts et al. [12], the result of our study indicate that a large weight retention at 6 weeks postpartum was associated with an higher risk of GDM, thus indicating a correlation between weight gain in the index pregnancy and GDM in the subsequent pregnancy.

Most published intervention trials which have focused upon reducing gestational weight gain in obese women were unable to decrease the increased perinatal risks [35, 36]. Consequently, the preconceptional period remains an important period when attempting to prevent an unhealthy maternal weight before the beginning of a pregnancy. The fact that we identified a strong association in weight gain between pregnancies and the development of GDM suggests that doctors should provide women with advice pertaining to weight change between pregnancies. The 6 weeks timepoint after an index pregnancy is a suitable and readily accessible follow-up point and provides a unique opportunity to not only identify risk, but to begin to implement primary preventative managements. In view of the fact that maintaining a stable BMI between pregnancies may reduce the risk of GDM in a subsequent pregnancy and according to the association between weight retention at 6 weeks postpartum and GDM in the second pregnancy, we could consider 6 weeks after the index pregnancy as the beginning of the next pregnancy. Using this timepoint, we could then identify high-risk groups, and establish individualized weight managements strategies with which to follow-up. The 6 weeks postpartum timepoint provides a new early window of opportunity to identify risk factors for a subsequent pregnancy and allows us to implement primary preventative strategies.

### Strength and limitation

We conducted a retrospective cohort study in a large population of patients and reported that weight retention at 6 weeks postpartum is associated with interpregnancy BMI change and GDM in the subsequent pregnancy. However, there are some limitations to our study that need consideration. In this study, participants were all Asian women; thus, the effect of lifestyle and dietary habit on PPWR and GDM might be different with other races. Furthermore, we did not include information relating to maternal education or smoking behavior. We were only able to control for maternal age. Another potential confounder is that we were not able to control the increased weight at the end of pregnancy resulting from an excess of amniotic fluid, which is often seen in GDM women.

## Conclusions

We demonstrated that weight retention between the first and second pregnancy increased the risk of GDM. A large weight retention at 6 weeks postpartum after an index pregnancy was significantly related to weight gain between pregnancies and GDM in the subsequent pregnancy. Taken together, the evidence indicates that women require a more intensive period follow-up during the postpartum period.

## Data Availability

The datasets used and/or analyzed during the current study are available from the corresponding author on reasonable request.

## Abbreviations

BMI: Body mass index
CIs: Confidence intervals
GDM: Gestational diabetes mellitus
OR: Odds ratio
